# Results of an expert Delphi consensus from the Italian Society of Medical and Interventional Radiology (SIRM) and the Italian Society of Rheumatology (SIR) on standardized requesting and reporting magnetic resonance imaging in patients with suspected or known axial spondyloarthritis

**DOI:** 10.1007/s11547-025-02088-7

**Published:** 2025-09-30

**Authors:** Fausto Salaffi, Marina Carotti, Fabio Martino, Emilio Filippucci, Sonia Farah, Roberta Ramonda, Andrea Doria, Roberto Caporali, Marcello Govoni, Piercarlo Sarzi-Puttini, Alberto Batticiotto, Luca Ceccarelli, Maurizio Rossini, Enrico Scarano, Luca Maria Sconfienza, Stefania Vio, Carlo Masciocchi, Alessandro Muda, Ernesto La Paglia, Massimo De Filippo, Marcello Zappia, Mauro Battista Gallazzi, Salvatore D’Angelo, Francesca Oliviero, Marco Di Carlo, Cristiana Barreca, Marco Canzoni, Enzo Silvestri, Alberto Aliprandi, Antonio Barile, Alessandra Splendiani, Andrea Giovagnoni, Antonio Leone, Nicoletta Gandolfo

**Affiliations:** 1 Rheumatology Unit, Università Politecnica delle Marche, “Carlo Urbani” Hospital, Via Aldo Moro, 25, 60035 Jesi, Ancona Italy; 2 Department of Radiological Sciences - Division of Clinical Radiology, University Hospital Azienda Ospedaliero Universitaria delle Marche, Ancona, Italy; 3 Sant’Agata Radiology Center, Bari, Italy; 4 Rheumatology Unit, Department of Medicine (DIMED), Padova University Hospital, Padua, Italy; 5 Department of Rheumatology and Medical Sciences, ASST Gaetano Pini-CTO, Milan, Italy; 6 Rheumatology Unit, Azienda Ospedaliero-Universitaria S. Anna, Department of Medical Sciences, University of Ferrara, Ferrara, Italy; 7 Department of Biomedical and Clinical Sciences, University of Milan, Milan, Italy; 8 IRCCS Ospedale Galeazzi Sant’Ambrogio, Milan, Italy; 9 Rheumatology Unit, Internal Medicine Department, ASST Sette Laghi, Ospedale di Circolo, Fondazione Macchi, Varese, Italy; 10 Pediatric and Adult Cardiothoracic and Vascular, Oncohematologic and Emergency Radiology Unit, IRCCS Azienda Ospedaliero-Universitaria di Bologna, Bologna, Italy; 11 Rheumatology Unit, Department of Medicine, University of Verona, Verona, Italy; 12 Department of Radiology, “San Carlo” Hospital, Potenza, Italy; 13 Department of Biomedical Sciences for Health, University of Milan, IRCCS Ospedale Galeazzi Sant’Ambrogio, Milan, Italy; 14 Radiology Unit II, Integrated Complex, Azienda Ospedaliera - University Hospital of Padua, Padua, Italy; 15 Department of Biotechnological and Applied Clinical Sciences, University of L’Aquila, L’Aquila, Italy; 16 Section of Radiology, Department of Radiology, IRCCS San Martino University Hospital, University of Genoa, Genoa, Italy; 17 Unit of Diagnostic Imaging, Humanitas Cellini, Turin, Italy; 18 Department of Medicine and Surgery, Section of Radiology, University of Parma, Maggiore Hospital, Parma, Italy; 19 Department of Medicine and Health Sciences, University of Molise, Campobasso, Italy; 20 Department of Radiology, ASST Gaetano Pini-CTO Orthopedic and Traumatology Specialist Center, Milan, Italy; 21 Department of Health Science, University of Basilicata, Rheumatology Unit, San Carlo Hospital, Potenza, Italy; 22 Section of Rheumatology, Department of Medical, Surgical, and Neurological Sciences, Santa Maria Alle Scotte Hospital, Siena, Italy; 23 Simple Departmental Operational Unit Rheumatology (UOSD), Nuovo Regina Margherita Hospital ASL Roma-1, 00153 Rome, Italy; 24 Department of Imaging Diagnostics, Salus Institute – Alliance Medical, Genoa, Italy; 25 Department of Radiology and Diagnostic Imaging, Istituti Clinici Zucchi Monza, University of Milano-Bicocca, Milan, Italy; 26 Catholic University of the Sacred Heart of Rome, Rome, Italy; 27 Diagnostic Imaging Department, Villa Scassi Hospital-ASL 3, Genoa, Italy

**Keywords:** Axial spondyloarthritis, Radiologists, Rheumatologists, Magnetic resonance imaging, Delphi consensus

## Abstract

**Objectives:**

To develop a practical consensus for standardizing communication between rheumatologists and radiologists regarding magnetic resonance imaging (MRI) of the sacroiliac joints and spine in the diagnosis and management of axial spondyloarthritis (axSpA).

**Methods:**

A task force comprising six rheumatologists and five musculoskeletal radiologists with expertise in axSpA imaging reviewed the Assessment of SpondyloArthritis International Society (ASAS) framework to draft initial recommendations and define project goals. A broader expert panel (21 rheumatologists, 19 radiologists) then participated in a voting process to refine and validate these recommendations. Final endorsement was sought from the steering committees of the Italian Society of Medical and Interventional Radiology (SIRM) and the Italian Society of Rheumatology (SIR) using a modified Delphi method.

**Results:**

Thirty-one recommendations were validated, organized into eight domains. Domain 1 outlines five overarching principles. Domain 2 comprises recommendations on clinical features, symptoms, and risk factors. Additional domains address MRI technical parameters, including image quality and sequencing (Domain 3), and standardized reporting criteria. For the sacroiliac joints (Domains 4 and 5), five signs of inflammation and six of structural damage are defined. For the spine (Domains 6 and 7), five inflammatory and four structural features are specified. Domain 8 provides guidance on report conclusions. The recommendations were endorsed by SIRM/SIR with 88.5% approval.

**Conclusion:**

This consensus offers structured guidance for MRI requesting and reporting in axSpA, fostering clear communication between radiologists and rheumatologists. The standardized approach aims to improve diagnostic accuracy and patient outcomes.

## Introduction

The term axial spondyloarthritis (axSpA) refers to a group of chronic inflammatory rheumatic diseases with a shared genetic background, characterized by enthesitis and both axial and peripheral joint involvement [1]. One of the major challenges in managing axSpA is the considerable diagnostic delay, which typically averages 8–9 years [2]. This delay persists despite ongoing symptoms of inflammatory back pain (IBP) and is largely attributable to the slow progression of radiographic changes in the sacroiliac joints (SIJs), the limited sensitivity of laboratory tests, and the nonspecific nature of early clinical signs. The social burden of delayed axSpA diagnosis is also substantial, largely due to productivity loss [3]. These challenges prompted the Assessment of SpondyloArthritis International Society (ASAS) to conduct a comprehensive reevaluation of SpA classification criteria. Through a series of data-driven studies, ASAS demonstrated that magnetic resonance imaging (MRI) could significantly improve patient stratification by reclassifying individuals presenting with back pain into more accurate diagnostic categories [4]. Consequently, MRI was incorporated as an imaging criterion in the updated classification framework for axSpA (5). According to ASAS-defined criteria, baseline MRI demonstrates strong diagnostic performance for axSpA, as confirmed by concordant reader data. The utility of MRI in the diagnosis of axSpA, compared to expert opinion, shows a positive likelihood ratio of 11.8 (with a sensitivity of 66% and a specificity of 94%, respectively). Moreover, the sensitivity of MRI in predicting the subsequent development of radiographic sacroiliitis is 100% [5].

Given the variability in MRI accessibility and the limited familiarity with axSpA imaging in some clinical settings, a collaborative effort among radiologists and rheumatologists aimed to develop practical consensus for the appropriate use of MRI in axSpA. It became evident that a standardized, reliable definition of a “positive MRI” for sacroiliitis and spondylitis was crucial for the broader application of these criteria in both clinical practice and research. To address this, a working group from ASAS and Outcome Measures in Rheumatology (OMERACT) reached a consensus on defining MRI positivity [6–8].

This consensus outlines precise criteria for identifying inflammatory lesions (sacroiliitis and spondylitis) and structural changes (e.g., fat deposition), providing descriptions of disease-related abnormalities in the SIJs and spine. These definitions can be consistently applied in both research contexts and routine clinical evaluations of patients with axSpA.

Specifically, “active sacroiliitis on MRI” is defined by the presence of synovitis, enthesitis, capsulitis, and bone marrow edema (BME)/osteitis—with BME/osteitis being essential for confirming active inflammation. MRI can also detect structural lesions such as ankylosis, sclerosis, erosions, and fat metaplasia. However, the diagnostic and classificatory significance of these structural lesions remains less well defined, particularly when they are subtle. In the spine, six types of inflammatory lesions have been described: spondylodiscitis, zygapophyseal joint arthritis, anterior and posterior spondylitis, and enthesitis of the spinal ligaments. Four types of structural lesions have also been identified: fatty deposition, erosions, syndesmophytes, and ankylosis. Among these, anterior/posterior spondylitis and fat deposition at vertebral corners are considered hallmark features of axSpA. Based on the literature and expert consensus, a spinal MRI is considered positive for inflammation when anterior/posterior spondylitis is present at three or more sites. In young adults, fat deposition at multiple vertebral corners is particularly suggestive of axSpA.

To improve MRI reporting in axSpA, ASAS has published specific guidelines for radiological interpretation [9]. The currently available guidelines have been developed within international forums, while no consensus documents contextualized to the Italian setting are presently available. Based on these considerations, the primary objective of this study is to develop a consensus document aimed at bridging the communication gap between radiologists, who interpret imaging studies, and rheumatologists, who request them, in the context of suspected or confirmed axSpA, with adaptability to the Italian healthcare setting.

## Methods

A modified Delphi method [10] was conducted in four stages between June 2024 and March 2025:

- 1. Formation of a steering committee task force composed of radiologists and rheumatologists;
- 2. Review of the ASAS statements and development of new items tailored to the Italian healthcare context, emphasizing the key role of sacroiliac joint and spinal imaging as facilitators of diagnosis and management;
- 3. Online voting on the newly proposed statements;
- 4. Evaluation, revision, and potential re-voting on any statements that did not reach consensus.

The objective of the process was to identify essential components for imaging recommendations and refine them for practical implementation [10]. The first in-person meeting was held in Milan on June 28, 2024. The task force consisted of six rheumatologists with expertise in axSpA imaging and five musculoskeletal radiologists specialized in inflammatory musculoskeletal diseases. Members were selected based on their publication records and previous engagement in related initiatives. After reviewing and discussing the ASAS framework, the task force developed a preliminary set of recommendations. Subsequently, a broader group of 19 radiologists and 21 rheumatologists from various regions of Italy—selected to reflect potential regional differences in clinical practice—were invited to participate in a decisive round of voting. These participants were identified through consensus among the steering committee members and in consultation with two national scientific societies: the Italian Society of Medical and Interventional Radiology (SIRM) and the Italian Society of Rheumatology (SIR), based on their professional contributions to the field. Each proposed statement was evaluated for its relevance in real-world clinical practice, with special attention to the following aspects:

1. Critical challenges and barriers to implementation;
2. Proposals to improve applicability;
3. The potential role of digital health in facilitating adoption.

All the invited members were asked to complete an online survey, rating their agreement with the 33 proposed statements using a scale from 0 (no agreement) to 10 (complete agreement). A statement was considered validated if it met two criteria: (1) a mean score of ≥ 8 and (2) at least 75% of responses scoring ≥ 8. These dual thresholds were adopted to account for possible skewed vote distributions or the presence of strongly dissenting opinions, ensuring a more robust consensus process than using a single metric alone.

Following the survey, the steering committee revised the statements as needed and finalized the wording. The results of the final survey were reviewed and finalized during a consensus meeting held via teleconference on January 10, 2025. Upon achieving full agreement, a final consensus document was drafted. A summary of the main findings, along with the final statements and their corresponding agreement percentages, is presented below.

## Results

A total of 33 comprehensive recommendations were developed to guide clinicians in the imaging referral process for patients with suspected or confirmed axSpA. These recommendations cover clinical features suggestive of axSpA, relevant medical history, prior imaging, potential contraindications for specific imaging modalities or contrast agents, and the rationale for requesting imaging. Of the 33 statements, 31 ( 93.94%) achieved a mean agreement score of ≥ 8/10 and met the additional criterion of at least 75% of responses scoring ≥ 8. One general principle statement and one regarding MRI findings of structural damage in the SIJs did not meet these thresholds. The statements (validated and not) included in the online survey are presented in Table 1.

**Table 1:**
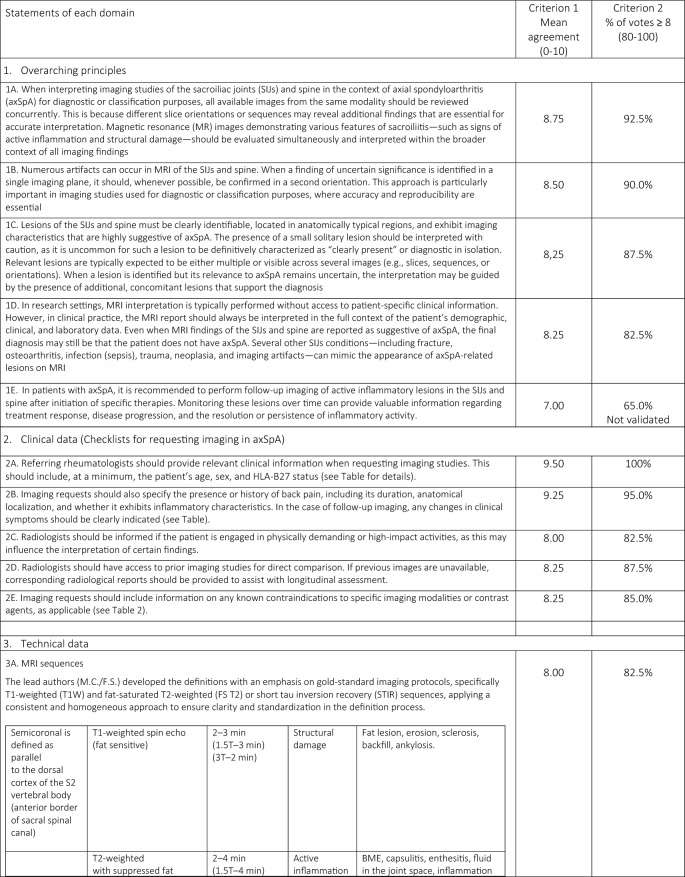

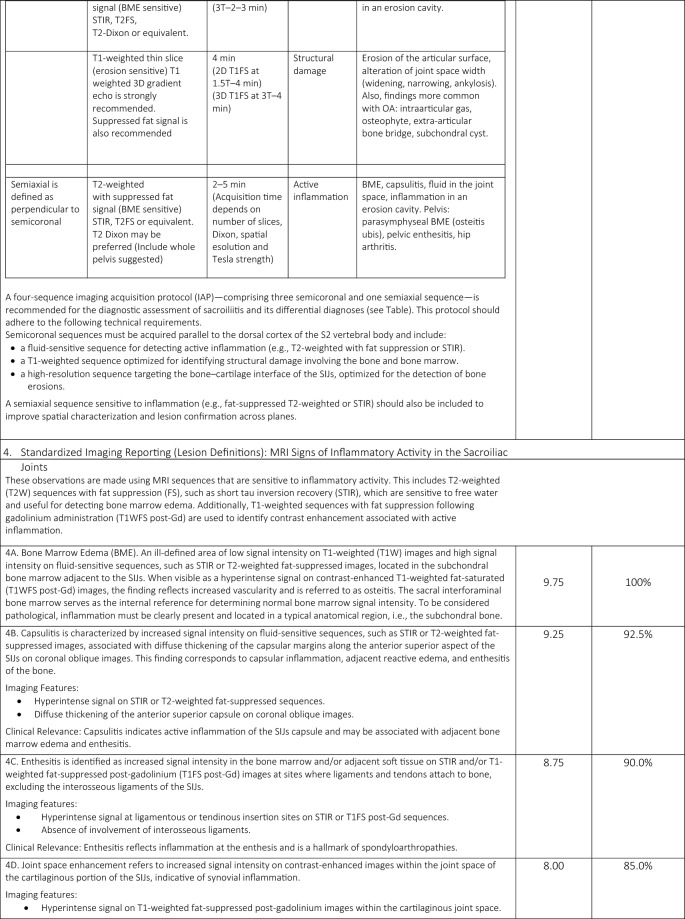

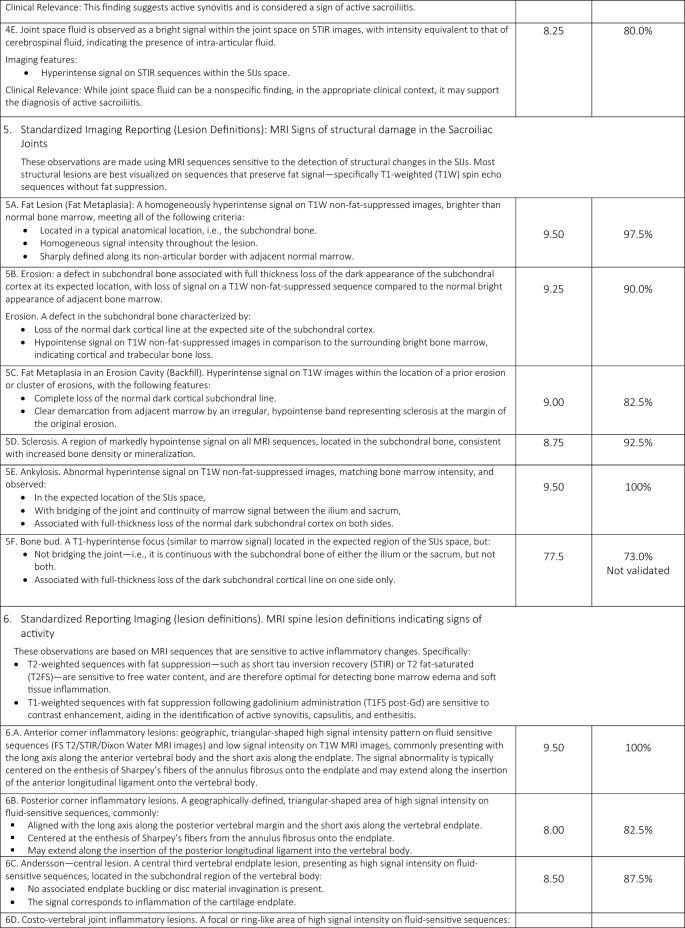

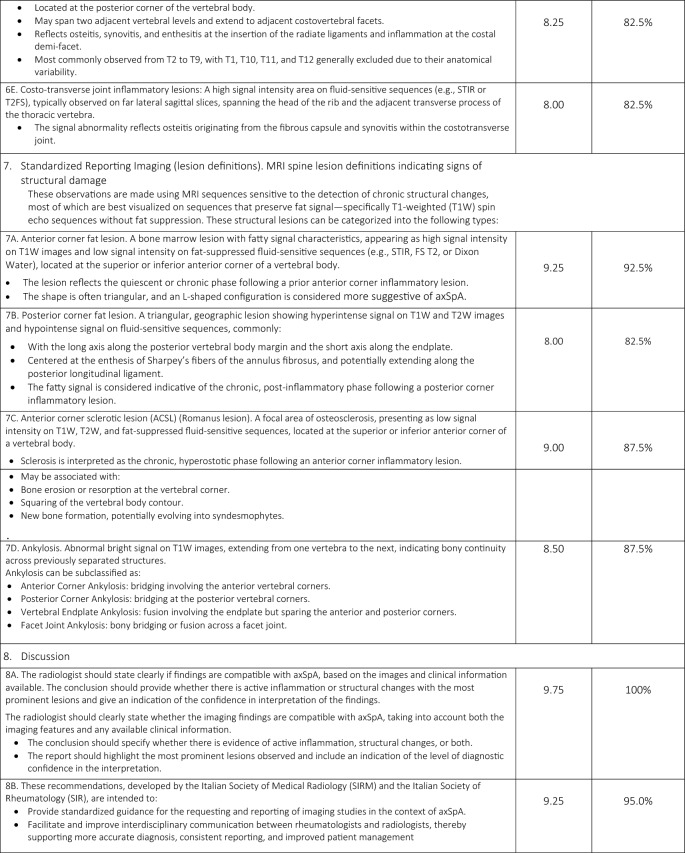
Final consensus on the applicability of the Italian expert group of 19 radiologists and 21 rheumatologists and level of agreement with the statements

A summary of the task force’s internal discussions on the applicability of ASAS statements to the Italian healthcare system and radiology/rheumatology practice is provided below, along with commentary on the rationale behind new proposed statements aimed at enhancing ASAS implementation in Italy.

### Domain 1: overarching principles

Consensus was reached on the importance of considering all available data—including active inflammatory and structural lesions—when interpreting SIJs and spinal MRI in the context of axSpA (Statement 1A). However, Statement 1E narrowly met the minimum validation threshold, with only 65% of respondents scoring ≥ 7/10, reflecting some divergence in expert opinion.

### Domain 2: communication of clinical information

The panel emphasized the need for effective communication between the referring rheumatologist and the radiologist, including comprehensive clinical data (e.g., age, sex, HLA-B27 status, symptom profile, physical activity level, and previous imaging). A checklist was developed (Table 2) to standardize referrals. Statement 2C achieved the minimum validation threshold, with noted barriers including limited consultation time, lack of communication training among rheumatologists, and inadequate use of adherence assessment tools.

**Table 2:**
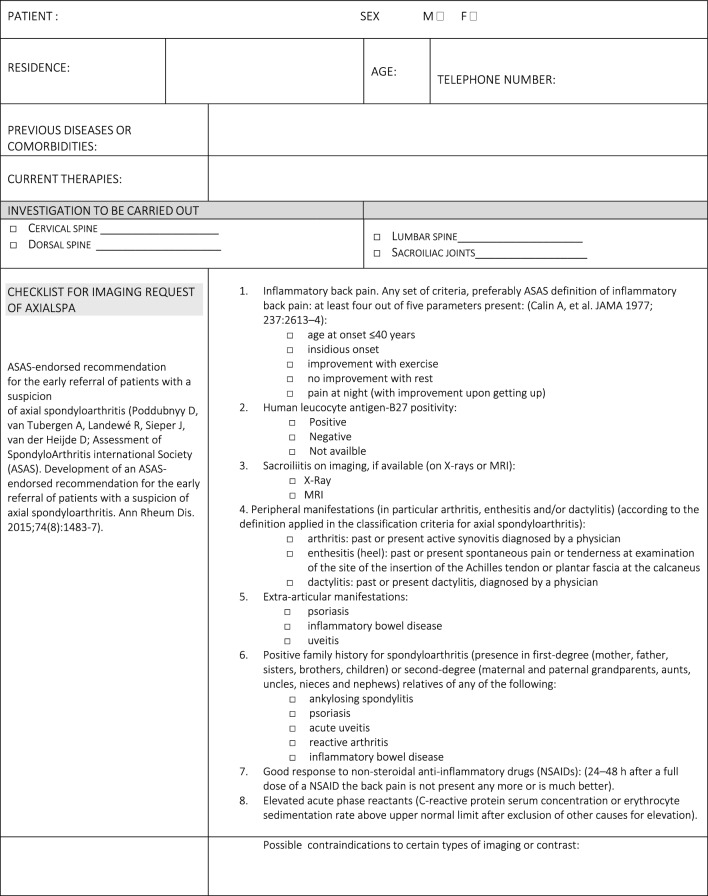
Checklist for communication of clinical information

### Domain 3: MRI protocol standardization

A standardized imaging acquisition protocol (IAP) including at least four sequences (3-semicoronal and 1-semiaxial) across two imaging planes was recommended for diagnostic ascertainment of sacroiliitis and its differential diagnoses. The European Society of Skeletal Radiology’s Arthritis Subcommittee released its guidelines for a SIJs MRI IAP in 2015, which called for four MRI sequences for diagnostic reasons [11]. Although validated, the statement achieved relatively lower agreement (mean score of 8, with 82.5% scoring ≥ 8/10), reflecting concerns about training, access, and implementation. Definitions and an atlas of inflammatory and structural lesions of the spine were created by the creators of the Canada-Denmark (CANDEN) MRI working group in 2009 [12, 13]. They also reported on the reliability of scoring the individual lesions in a multireader exercise [14]. The CANDEN technique makes it possible to systematically evaluate spine MRI from the standpoint of diagnostic determination and to quantify structural and active inflammatory abnormalities based on their exact anatomical positions and their relationships across time [15]. Short Tau Inversion Recovery (STIR) and sagittal T1-weighted (T1W) MRI sequences of the vertebral bodies and the posterior components of the vertebrae (i.e., the costotransverse, costovertebral and facet joints, transverse and spinous processes, and the surrounding soft tissues) served as the basis for the definitions.

### Domain 4: MRI sacroiliac joint lesion definitions indicating signs of activity

The panel agreed that accurate identification of BME requires strong T2-weighting and fat suppression, while contrast-enhanced sequences offer minimal additional value [16]. For the interpretation of MRI of the inflammatory SIJs lesions in the diagnosis of axSpA in the UK, the British Society of Spondyloarthritis issued a study of the subject in 2019 with seven suggestions [17]. Some patients with non-radiographic axSpA (nr-axSpA) do not exhibit MRI signs of inflammation, nevertheless, and this can also happen nonspecifically [18]. Proper T2-weighting of the necessary BME-sensitive sequence is necessary. This is not unexpected because, at most ages, sacral bone marrow is made up of both fatty and erythropoietic marrow. The erythropoietic marrow may appear relatively bright on a dark background when sequences are fat suppressed; however, this issue can be resolved with appropriate T2-weighting. T2-weighting has the effect of weakening the signal in bound water (like cartilage, muscle, or erythropoietic marrow), while leaving the signal strong in free water (like cerebrospinal fluid or BME). From a technical standpoint, this implies that the echo period must be sufficiently lengthy, roughly 80 + ms for spin echo sequences and 50 + ms for STIR. When compared to STIR or T2 with fat saturation, the water component of T2-weighted Dixon imaging seems to be just as successful at detecting BME [19]. Strong T2-weighting and the reduction or eradication of the bone marrow fat signal are essential components of the suggested strategy. Every author concurred that there are enough data to support the idea that contrast-enhanced sequences are typically not required in adults or children and ought to be saved for exceptional situations. With little added value, contrast enhancement would come with extra expense and difficulty. Furthermore, contrast enhancement of inflammatory tissue in the joint space and synovitis, which may be easier to discern with contrast enhancement, hardly ever occurs in adults without accompanying BME [20, 21]. In order to accurately show anatomy, visualize particular lesions, and diagnose sacroiliitis in all patients—not just those with axSpA—a T1W spin echo (T1WSE) sequence is thought to be necessary. Fat lesions and backfill in subchondral bone, which are significant axSpA-related lesions, are best visible on T1WSE sequences because they are "fat-sensitive." Any sequence acquisition can be divided into distinct fat-only and water-only pictures using the Dixon approach. According to Özgen, a T2-weighted multipoint Dixon sequence has a better contrast-to-noise ratio than traditional sequences and can show the BME, sclerosis, and fat lesions of sacroiliitis [20]. Nevertheless, neither erosion detection nor diagnosis accuracy was evaluated in the publication. While Chien et al. found that the presence of subchondral edema in active sacroiliitis reduced the diagnostic accuracy of SIJs erosion detection on T2W Dixon MRI [22], Athira et al. suggest that T2 Dixon sequences are superior or equivalent to conventional MRI sequences [21]. The fact that these investigations primarily addressed the MRI results of axSpA and included a small number of individuals severely limited them. The accuracy of T2-Dixon imaging against traditional sequences for a variety of SIJs disorders in larger populations of patients with low back pain is not yet compared in any publication. Therefore, it is not now possible to provide a substitute for incorporating a T1WSE.

### Domain 5: MRI sacroiliac joint lesion definitions indicating signs of structural damage

In early axSpA, there is a predictive and longitudinal correlation between inflammation found on MRI and the emergence of structural damage on MRI in the spine (fatty lesions) and SIJs (fatty lesions and erosions) over a 5-year period [23]. Because it supports its use for prognostic stratification, evidence showing inflammation on MRI induces structural damage in early axSpA is pertinent to practicing rheumatologists [24]. A number of structural MRI abnormalities in the SIJs have been reported in earlier research [25]. When radiographs are normal or ambiguous, MRI of the SIJs may show structural abnormalities, especially erosions, in individuals with nr-axSpA [26]. When the cortical bone of the iliac or sacral bones is breached, it appears dark on both T1WSE and STIR sequences. Additionally, on T1WSE MRI, the strong signal from the surrounding marrow matrix is gone, indicating the presence of erosion [27]. A lesion in bone marrow close to subchondral bone may also be observed on the T1WSE MRI scan. This lesion is differentiated by a consistent rise in marrow signal, which is a hallmark of lipid accumulation, and a visible boundary [28]. This lesion’s histopathologic nature is still unknown; it might represent adipose tissue, but it could also reflect lipid accumulation in distinct cell types, which can happen as a cell matures into a different phenotype. This lesion has been called fat metaplasia because it has recently been established to indicate tissue modification after BME clearance [25, 29]. Similar tissue has been shown to form at erosion locations after inflammation has subsided; this phenomenon has been dubbed "backfill." The reparative fatty metaplasia adjacent to the SIJs, which are encircled by sclerosis, is known as the "backfill," an intermediate stage between erosion and ankylosis [25]. Together with a semi-quantitative scoring system that evaluates each unique lesion, standardized classifications for these various lesions have been created and validated [28]. The significance of standardizing an imaging report to best define MRI sacroiliac joint lesions exhibiting evidence of structural deterioration was nearly universally agreed upon, much like the prior assertion. The primary barriers to including MRI as a crucial component of the accepted diagnostic procedure for patients with axSpA are the quality of educational programs and the absence of specialized training for radiologists. Statement 5F bone bud met the minimum validation threshold, with only 73% of respondents scoring ≥ 7/10, reflecting some divergence in expert opinion.

### Domain 6: role of MRI spine lesion indicating signs of activity

MRI’s value in detecting early inflammatory spinal lesions, particularly in nr-axSpA, was affirmed. The use of spine MRI has grown since that 2012 article [8], and knowledge of how to identify and interpret structural and inflammatory spinal abnormalities in relation to clinical symptoms in axSpA and differential diagnosis has greatly improved. Since MRI can identify inflammatory spinal lesions in patients with early-onset axSpA who do not exhibit radiographic sacroiliitis, it is a valuable supplementary screening tool [7]. The ASAS/OMERACT working group defined a positive spinal MRI scan as having BME next to the vertebral endplates at the point where the anterior and posterior longitudinal ligaments insert into the facet joints and the annulus fibrosus attaches to the vertebral rim [30]. T2-weighted sequences with fat suppression that are sensitive for free water, like STIR, or T2FS or T1W sequences with fat suppression that are sensitive for contrast enhancement, like T1FS post-Gd, are examples of MRI sequences that are sensitive for the detection of disease activity. MRI is frequently used in clinical practice because of its great sensitivity in identifying inflammatory lesions of the spine, particularly when evaluating patients who may have axSpA and/or a history of persistent inflammatory low back pain [8, 31]. Other lesions affecting the vertebral bodies include the thoracic lateral inflammatory lesion (a lesion located posteriorly in a lateral slice, also known as arthritis of the costovertebral joints), which is only documented for the thoracic spine, and the vertebral endplate inflammatory lesion (also known as aseptic spondylodiscitis or Andersson—central lesion). Increased signal in bone marrow in at least one sagittal slice in a water-sensitive sequence in one of the other posterior elements where there are ligamentous or muscular attachments, or at the costotransverse joint (excluding the pedicle, facet processes, and pars interarticularis) is indicative of an inflammatory lesion of the posterior elements, which includes enthesitis of the spinal ligaments and inflammation of the costotransverse joint. The experts agreed that patients are more likely to be motivated and eager to follow treatment suggestions when they receive care that is customized to their preferences and goals. Statements 6D and 6E met only the minimum consensus threshold, possibly due to differences in interpretation and technological limitations.

### Domain 7: role of MRI spine lesion indicating signs of structural damage

When typical features such as fat lesions, erosions, sclerosis, syndesmophytes, or ankylosis are clearly present at the vertebrae, they are referred to as structural lesions. Any kind of structural lesion may appear alone or in conjunction with or encircled by BME. Only on sequences that are sensitive to fat signals, specifically T1WSE without fat suppression, can the majority of the data be clearly detected. While the concept of fat lesions was the same, it was thought that erosions, syndesmophytes, and ankylosis needed an update on structural lesions [32, 33]. The MRI sequences used for these observations are sensitive enough to identify structural changes. Only sequences that are sensitive to fat signals, specifically T1WSE without fat suppression, may clearly display the majority of the observations. These can be separated into:

1. Bone erosion occurs when at least one sagittal slice of T1W images shows a full-thickness loss of the black look of cortical bone and a loss of the typical bright appearance of neighboring bone marrow. The only erosions evaluated are those that affect the vertebral corners. There are two types of erosions: anterior and posterior corner erosions;
2. Focal fat lesion: At least two sagittal slices of T1W images show a focally elevated signal in the bone marrow. Only fat lesions that affect the corners of the vertebrae are evaluated. There are two types of fat lesions: anterior and posterior corner fat lesions;
3. Bone spur toward the anterior or posterior longitudinal ligament (also called syndesmophytes): in at least one sagittal slice, a bright signal on T1W images that extends vertically from the vertebral corner to the neighboring vertebral corner. Bone spurs are separated into anterior and posterior corner bone spurs, which are found in the anterior and posterior corners, respectively, and do not extend to the neighboring vertebra;
4. On T1W images, ankylosis is characterized by a strong signal that extends from a vertebra and is continuous with the neighboring vertebra. The anterior and posterior corner ankylosis (found in the anterior and posterior corners, respectively) is the two subtypes of this. Ankylosis affecting the endplate but not the anterior or posterior vertebral corners is known as vertebral endplate ankylosis;
5. Ankylosis of a facet joint is known as facet joint ankylosis.

The experts concurred that any of the above-mentioned structural lesions at the location of a degenerating disk lesion should not be taken as a sign of axSpA. The specialists also underlined that bone spurs, which are found in the longitudinal ligaments and the tissue that is closely connected to them, are known to be harder to find on a traditional MRI than on a traditional radiograph or computed tomography (CT) scan. Additionally, the presence of syndesmophytes may not always be taken as a trustworthy indicator of axSpA, particularly when it comes to the identification on MRI as opposed to the identification on CT or conventional radiographs [34]. Furthermore, experts feel that regular remote patient completion of validated adherence questionnaires and frequent healthcare providers monitoring of such data can encourage a deeper and faster knowledge of the patient's genuine adherence (6E). Every statement that was prepared and subsequently placed up for online voting satisfied the conditions for validation. It should be highlighted, meanwhile, that statements 6D and 6E only exceeded the second criterion's minimum threshold value (75% of responses equal to or greater than 8/10) because some panelists gave them lower scores.

### Domain 8: final considerations and future directions

The definitions for SIJs and spinal MRI abnormalities of patients referred with unidentified back pain and clinical suspicion of axSpA have been updated based on consensus in this publication. In addition to supporting the finding that BME and fat lesions play a significant role in identifying pathologic findings when analyzing spinal MRIs, these results could also be utilized in continuing attempts to reevaluate what constitutes a "positive" MRI of the spine in relation to axSpA versus non-axSpA. With a relatively high proportion of unanimity, both of the supplementary statements have mostly met the validation criteria.

## Discussion

To enhance the quality of clinical information included in imaging referrals for suspected or confirmed axSpA, the ASAS has developed a series of important recommendations [2]. This initiative builds upon previous ASAS work, particularly in the areas of imaging acquisition protocols [35], interpretation standards [32, 36], reporting guidelines [9], and broader management strategies for patients with axSpA [2]. The overarching aim of these guidelines is to strengthen collaborative communication among the various medical professionals involved in the diagnosis and care of axSpA patients—namely rheumatologists, radiologists, and imaging technicians. The recommendations align with current diagnostic workflows and decision-making frameworks used in the evaluation of patients with suspected axSpA. The development process was supported by active discussions among radiologists and rheumatologists, which contributed a range of perspectives and helped shape the final consensus. In the sections above, we outlined key areas of agreement as well as some points of contention that emerged during the task force meetings and workshop deliberations.

This consensus is not without limitations. First, the recommendations are expert-driven and rooted in clinical experience and practical preferences rather than population-based data. While certain elements—such as including patient age, sex, and contraindications in referral forms—may appear self-evident or already reflected in national protocols, the SIRM/SIR collaborative group recognizes that healthcare infrastructure and procedural norms can vary significantly across Italian healthcare settings and regions. Second, the scope of this initiative was limited to adult patients with suspected or confirmed axSpA. As such, the recommendations may not be directly applicable to younger populations, who often require tailored imaging strategies and clinical considerations. Lastly, although these statements may place additional demands on referring rheumatologists in terms of documentation and communication, their primary intent is to foster a more reciprocal and effective exchange of clinical information between specialties. Improved communication between rheumatology and radiology teams is essential to optimizing diagnostic accuracy and patient care.
