# Correction: Results of an expert Delphi consensus from the Italian Society of Medical and Interventional Radiology (SIRM) and the Italian Society of Rheumatology (SIR) on standardized requesting and reporting magnetic resonance imaging in patients with suspected or known axial spondyloarthritis

**DOI:** 10.1007/s11547-025-02146-0

**Published:** 2025-11-04

**Authors:** Fausto Salaffi, Marina Carotti, Fabio Martino, Emilio Filippucci, Sonia Farah, Roberta Ramonda, Andrea Doria, Roberto Caporali, Marcello Govoni, Piercarlo Sarzi-Puttini, Alberto Batticiotto, Luca Ceccarelli, Maurizio Rossini, Enrico Scarano, Luca Maria Sconfienza, Stefania Vio, Carlo Masciocchi, Alessandro Muda, Ernesto La Paglia, Massimo De Filippo, Marcello Zappia, Mauro Battista Gallazzi, Salvatore D’Angelo, Francesca Oliviero, Marco Di Carlo, Cristiana Barreca, Marco Canzoni, Enzo Silvestri, Alberto Aliprandi, Antonio Barile, Alessandra Splendiani, Andrea Giovagnoni, Antonio Leone, Nicoletta Gandolfo

**Affiliations:** 1 Rheumatology Unit, Università Politecnica delle Marche, “Carlo Urbani” Hospital, Via Aldo Moro, 25, 60035 Jesi, Ancona Italy; 2 Department of Radiological Sciences - Division of Clinical Radiology, University Hospital Azienda Ospedaliero Universitaria delle Marche, Ancona, Italy; 3 Sant’Agata Radiology Center, Bari, Italy; 4 Rheumatology Unit, Department of Medicine (DIMED), Padova University Hospital, Padua, Italy; 5 Department of Rheumatology and Medical Sciences, ASST Gaetano Pini-CTO, Milan, Italy; 6 Rheumatology Unit, Azienda Ospedaliero-Universitaria S. Anna, Department of Medical Sciences, University of Ferrara, Ferrara, Italy; 7 Department of Biomedical and Clinical Sciences, University of Milan, Milan, Italy; 8 IRCCS Ospedale Galeazzi Sant’Ambrogio, Milan, Italy; 9 Rheumatology Unit, Internal Medicine Department, ASST Sette Laghi, Ospedale di Circolo, Fondazione Macchi, Varese, Italy; 10 Pediatric and Adult Cardiothoracic and Vascular, Oncohematologic and Emergency Radiology Unit, IRCCS Azienda Ospedaliero-Universitaria di Bologna, Bologna, Italy; 11 Rheumatology Unit, Department of Medicine, University of Verona, Verona, Italy; 12 Department of Radiology, “San Carlo” Hospital, Potenza, Italy; 13 Department of Biomedical Sciences for Health, University of Milan, IRCCS Ospedale Galeazzi Sant’Ambrogio, Milan, Italy; 14 Radiology Unit II, Integrated Complex, Azienda Ospedaliera - University Hospital of Padua, Padua, Italy; 15 Department of Biotechnological and Applied Clinical Sciences, University of L’Aquila, L’Aquila, Italy; 16 Section of Radiology, Department of Radiology, IRCCS San Martino University Hospital, University of Genoa, Genoa, Italy; 17 Unit of Diagnostic Imaging, Humanitas Cellini, Turin, Italy; 18 Department of Medicine and Surgery, Section of Radiology, University of Parma, Maggiore Hospital, Parma, Italy; 19 Department of Medicine and Health Sciences, University of Molise, Campobasso, Italy; 20 Department of Radiology, ASST Gaetano Pini-CTO Orthopedic and Traumatology Specialist Center, Milan, Italy; 21 Department of Health Science, University of Basilicata, Rheumatology Unit, San Carlo Hospital, Potenza, Italy; 22 Section of Rheumatology, Department of Medical, Surgical, and Neurological Sciences, Santa Maria Alle Scotte Hospital, Siena, Italy; 23 Simple Departmental Operational Unit Rheumatology (UOSD), Nuovo Regina Margherita Hospital ASL Roma-1, 00153 Rome, Italy; 24 Department of Imaging Diagnostics, Salus Institute – Alliance Medical, Genoa, Italy; 25 Department of Radiology and Diagnostic Imaging, Istituti Clinici Zucchi Monza, University of Milano-Bicocca, Milan, Italy; 26 Catholic University of the Sacred Heart of Rome, Rome, Italy; 27 Diagnostic Imaging Department, Villa Scassi Hospital-ASL 3, Genoa, Italy

**Correction to: La radiologia medica** 10.1007/s11547-025-02088-7

In this article, author would like to correct his name which was incorrectly published as “Marco Di Carlo Di Carlo”, it should have been published as “Marco Di Carlo”.

The original article has been corrected.

